# Supplementary figures and images for: Transition in social risk factors and adolescent motherhood in low- income and middle- income countries: Evidence from Demographic and Health Survey data, 1996–2018
Source: PLOS Glob Public Health. 2022 May 11;2(5):e0000170. doi: 10.1371/journal.pgph.0000170 (PMC10021223; doi:10.1371/journal.pgph.0000170)

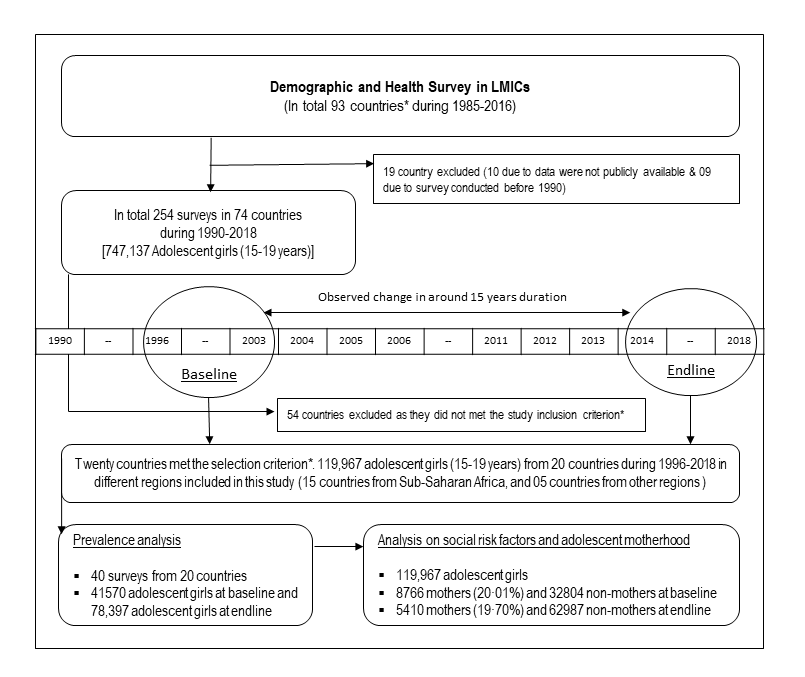

Supplement: S1 Fig — *The sample selection criterion was, country with at least two all-women surveys: a survey in 1996–2003, near to MDGs started (as baseline) and another survey in 2014–2018, recent time (as endline) so that the duration for assessing the transition of social factors can be nearly 15 years for all the studied countries. (TIF) [file pgph.0000170.s001.tif]

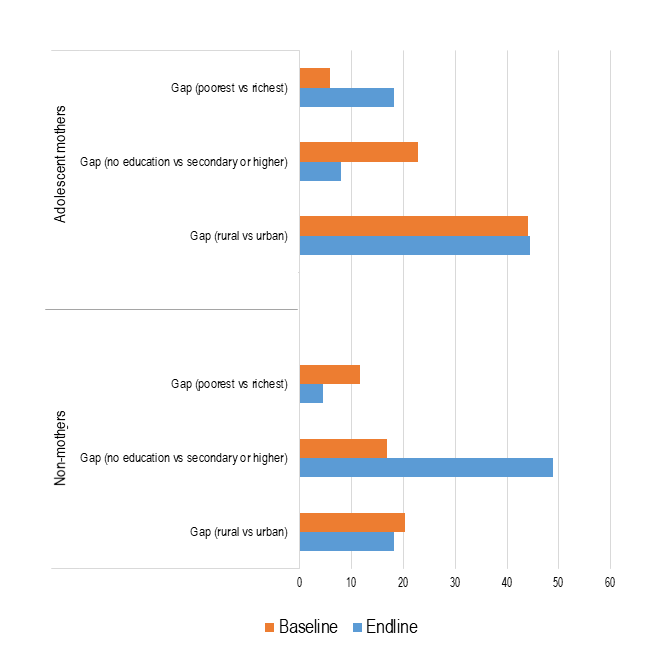

Supplement: S2 Fig — (TIF) [file pgph.0000170.s002.tif]
