# Supplementary material for: Transition in social risk factors and adolescent motherhood in low- income and middle- income countries: Evidence from Demographic and Health Survey data, 1996–2018
Source: PLOS Glob Public Health. 2022 May 11;2(5):e0000170. doi: 10.1371/journal.pgph.0000170 (PMC10021223; doi:10.1371/journal.pgph.0000170)
Supplement: S1 Table — *Difference in the prevalence between baseline and endline survey; CI: Confidence interval. (DOCX) [file pgph.0000170.s003.docx]

**S1 Table: List of survey countries, study participants (adolescent girls aged 15-19 years), weighted prevalence of adolescent motherhood.**

| **Country/Region name** | **Survey time** | **Survey year** | **No. of interviewed women** | **No. of adolescent girls (15-19 years)** | **Weighted prevalence in % (95%CI)** | **Difference in the prevalence (P-value)^*^** |
| --- | --- | --- | --- | --- | --- | --- |
| **sub-Saharan Africa** |  |  |  |  |  |  |
| Benin | Endline | 2017 | 15,928 | 3,335 | 20·07(18·75, 21·47) | -1·38(0·321) |
|  | Baseline | 2001 | 6,219 | 1,223 | 21·46(19·25, 23·84) |  |
| Chad | Endline | 2014 | 17,719 | 3,889 | 35·91(34·42, 37·42) | -2·59(0·096) |
|  | Baseline | 1996 | 7,454 | 1,716 | 38·5(36·22, 40·82) |  |
| Ethiopia | Endline | 2016 | 15,683 | 3,498 | 12·48(11·4, 13·63) | -3·78(0·002) |
|  | Baseline | 2000 | 15,367 | 3,584 | 16·26(15·11, 17·48) |  |
| Ghana | Endline | 2014 | 9,396 | 1,756 | 14·17(12·56, 15·95) | +0·09(0·954) |
|  | Baseline | 1998 | 4,843 | 889 | 14·08(11·96, 16·49) |  |
| Guinea | Endline | 2018 | 10,874 | 2561 | 25·96(24·31, 27·68) | -11·23(<0·0001) |
|  | Baseline | 1999 | 6,753 | 1,339 | 37·2(34·63, 39·84) |  |
| Kenya | Endline | 2014 | 31,079 | 6,078 | 18·09(17·13, 19·1) | -2·77(0·025) |
|  | Baseline | 1998 | 7,881 | 1,852 | 20·86(19·07, 22·77) |  |
| Malawi | Endline | 2015 | 24,562 | 5,273 | 29·03(27·82, 30·27) | -3·97(0·002) |
|  | Baseline | 2000 | 13,220 | 2,914 | 33(31·3, 34·74) |  |
| Mali | Endline | 2018 | 10,519 | 2,209 | 36(33·98, 38·08) | -4·44(0·009) |
|  | Baseline | 2001 | 12,849 | 2,542 | 40·44(38·56, 42·36) |  |
| Nigeria | Endline | 2018 | 41,821 | 8,423 | 18·73(17·92, 19·58) | -6·51(<0·0001) |
|  | Baseline | 2003 | 7,620 | 1,749 | 25·24(23·24, 27·36) |  |
| Rwanda | Endline | 2014 | 13,497 | 2,779 | 7·27(6·36, 8·3) | +0·43(0·57) |
|  | Baseline | 2000 | 10,421 | 2,727 | 6·84(5·94, 7·87) |  |
| Senegal | Endline | 2017 | 16,787 | 3,920 | 16·44(15·29, 17·67) | -5·83(<0·0001) |
|  | Baseline | 1997 | 8,593 | 1,958 | 22·28(20·48, 24·19) |  |
| South Africa | Endline | 2016 | 8,514 | 1,505 | 15·55(13·76, 17·53) | -0·41(0·771) |
|  | Baseline | 1998 | 11,735 | 2,373 | 15·97(14·51, 17·54) |  |
| Tanzania | Endline | 2015 | 13,266 | 2,932 | 26·67(25·09, 28·31) | +2·15(0·308) |
|  | Baseline | 1999 | 4,029 | 933 | 24·52(21·83, 27·42) |  |
| Uganda | Endline | 2016 | 18,506 | 4,276 | 24·79(23·52, 26·11) | -6·63(<0·0001) |
|  | Baseline | 2000 | 7,246 | 1,687 | 31·42(29·2, 33·73) |  |
| Zimbabwe | Endline | 2015 | 9,955 | 2,156 | 21·63(19·96, 23·4) | +1·15(0·442) |
|  | Baseline | 1999 | 5,907 | 1,468 | 20·48(18·47, 22·63) |  |
| **Other regions** |  |  |  |  |  |  |
| **South and southeast Asia** |  |  |  |  |  |  |
| Cambodia | Endline | 2014 | 17,578 | 3,006 | 11·99(10·85, 13·22) | +3·78(<0·0001) |
|  | Baseline | 2000 | 15,351 | 3,564 | 8·21(7·36, 9·15) |  |
| Philippines | Endline | 2017 | 25,074 | 5,120 | 8·56(7·81, 9·38) | +1·32(0·087) |
|  | Baseline | 1998 | 13,983 | 2,949 | 7·24(6·35, 8·23) |  |
| **Latin America & the Caribbean** |  |  |  |  |  |  |
| Colombia | Endline | 2015 | 38,718 | 6,604 | 17·36(16·43, 18·33) | -1·74(0·106) |
|  | Baseline | 2000 | 11,585 | 2,266 | 19·1(17·53, 20·77) |  |
| Guatemala | Endline | 2014 | 25,914 | 5,771 | 20·66(19·63, 21·72) | -0·96(0·571) |
|  | Baseline | 1998 | 6,021 | 1,403 | 21·62(19·49, 23·91) |  |
| Haiti | Endline | 2016 | 15,513 | 3,307 | 10·0 (9·0, 11·1) | -8·02(<0·0001) |
|  | Baseline | 2000 | 10,159 | 2,434 | 18·02(16·52, 19·63) |  |

^*^Difference in the prevalence between baseline and endline survey; CI: Confidence interval
