# Supplementary material for: Transition in social risk factors and adolescent motherhood in low- income and middle- income countries: Evidence from Demographic and Health Survey data, 1996–2018
Source: PLOS Glob Public Health. 2022 May 11;2(5):e0000170. doi: 10.1371/journal.pgph.0000170 (PMC10021223; doi:10.1371/journal.pgph.0000170)
Supplement: S2 Table — (DOCX) [file pgph.0000170.s004.docx]

**S2 Table: Measurement of variables used in this study**

| **Type of variable** | **List of variables** | **Description** | **Measurements/coding in this study** |
| --- | --- | --- | --- |
| Outcome variables | Adolescent motherhood | Women who have begun childbearing (either gave birth or first became pregnant) before age of 20 at the time of interview were considered as adolescent mothers in this study. | Nominal: Yes, No |
| Social risk factors | Wealth quantile | DHS splits all interviewed households into five wealth quintiles based on the estimated wealth index. The wealth index is calculated using principal component analysis on a household’s ownership of selected assets data, such as televisions and bicycles; materials used for housing construction; and types of water access and sanitation facilities. | Ordinal: Poorest, Poorer, Middle, Richer, Richest |
|  | Level of education | Highest education level attended. This is a standardized variable in DHS datasets providing level of education in the following categories: No education, Primary, Secondary, and Higher. | Ordinal: No education, Primary, Secondary or higher |
|  | Area of residence | Type of place of residence where the respondent was lived during interview as either urban or rural. | Nominal: Urban, Rural |
