# Supplementary material for: Transition in social risk factors and adolescent motherhood in low- income and middle- income countries: Evidence from Demographic and Health Survey data, 1996–2018
Source: PLOS Glob Public Health. 2022 May 11;2(5):e0000170. doi: 10.1371/journal.pgph.0000170 (PMC10021223; doi:10.1371/journal.pgph.0000170)
Supplement: S3 Table — AOR: Adjusted odds ratio; CI: Confidence interval; AOR>1 (<1) represents increased (decreased) in the association between adolescent mother and social risk factors at endline compared to baseline. (DOCX) [file pgph.0000170.s005.docx]

**S3 Table: Association between social factors and adolescent motherhood in LMICs, and changes in the association (AOR) during 1996-2018: country-specific estimates**

| **Country** | **Model 2: Adjusted model; AOR (95% CI)** | **Model 3: Adjusted (with interaction); AOR (95% CI)** | |
| --- | --- | --- | --- |
|  | **Social factor** | **Social factor** | **Social factor X Survey time** |
| **Benin** |  |  |  |
| Wealth quintile |  |  |  |
| - Poorest | 3·01(2·21,4·11) | 5·5(3·15,9·60) | 0·43(0·23,0·81) |
| - Poorer | 2·57(1·89,3·50) | 3·5(2·03,6·04) | 0·63(0·34,1·18) |
| - Middle | 2·84(2·10,3·83) | 4·64(2·71,7·94) | 0·49(0·26,0·91) |
| - Richer | 2·01(1·50,2·70) | 2·19(1·25,3·87) | 0·86(0·45,1·65) |
| - Richest | Ref | Ref | Ref |
| Level of education |  |  |  |
| - No education | 2·74(2·21,3·38) | 5·85(3·25,10·5) | 0·41(0·22,0·76) |
| - Primary | 1·43(1·14,1·78) | 2·66(1·41,5·01) | 0·52(0·27,1·02) |
| - Secondary or higher | Ref | Ref | Ref |
| Area of residence |  |  |  |
| - Rural | 1·26(1·03,1·54) | 2·1(1·46,3·02) | 0·58(0·38,0·87) |
| - Urban | Ref | Ref | Ref |
| **Cambodia** |  |  |  |
| Wealth quintile |  |  |  |
| - Poorest | 1·3(0·94,1·78) | 0·78(0·51,1·18) | 2·75(1·62,4·67) |
| - Poorer | 1·32(0·96,1·80) | 0·88(0·58,1·33) | 2·21(1·29,3·78) |
| - Middle | 1·36(1·00,1·86) | 0·97(0·64,1·46) | 1·96(1·14,3·37) |
| - Richer | 1·22(0·91,1·64) | 1·11(0·75,1·65) | 1·18(0·69,2·04) |
| - Richest | Ref | Ref | Ref |
| Level of education |  |  |  |
| - No education | 3·88(2·91,5·19) | 2·32(1·56,3·45) | 2·43(1·34,4·40) |
| - Primary | 2·09(1·69,2·58) | 1·26(0·88,1·81) | 1·97(1·29,3·00) |
| - Secondary or higher | Ref | Ref | Ref |
| Area of residence |  |  |  |
| - Rural | 1·23(0·95,1·59) | 0·93(0·66,1·31) | 1·82(1·19,2·81) |
| - Urban | Ref | Ref | Ref |
| **Chad** |  |  |  |
| Wealth quintile |  |  |  |
| - Poorest | 1·09(0·84,1·41) | 0·42(0·27,0·65) | 3·45(2·17,5·50) |
| - Poorer | 1·13(0·88,1·46) | 0·57(0·41,0·81) | 2·68(1·83,3·91) |
| - Middle | 0·87(0·68,1·12) | 0·71(0·49,1·03) | 1·28(0·85,1·93) |
| - Richer | 0·81(0·64,1·02) | 0·84(0·61,1·16) | 0·93(0·63,1·37) |
| - Richest | Ref | Ref | Ref |
| Level of education |  |  |  |
| - No education | 2·41(1·98,2·92) | 3·78(2·29,6·25) | 0·57(0·33,0·97) |
| - Primary | 1·9(1·56,2·33) | 3·09(1·86,5·13) | 0·55(0·32,0·95) |
| - Secondary or higher | Ref | Ref | Ref |
| Area of residence |  |  |  |
| - Rural | 1·14(0·92,1·42) | 0·76(0·58,0·99) | 1·55(1·14,2·11) |
| - Urban | Ref | Ref | Ref |
| **Colombia** |  |  |  |
| Wealth quintile |  |  |  |
| - Poorest | 5·32(3·96,7·16) | 4·06(2·61,6·30) | 1·5(0·87,2·58) |
| - Poorer | 4·02(3·09,5·23) | 3·47(2·31,5·20) | 1·29(0·76,2·22) |
| - Middle | 2·66(2·03,3·49) | 2·76(1·84,4·15) | 1(0·58,1·74) |
| - Richer | 1·41(1·05,1·90) | 1·26(0·80,1·97) | 1·24(0·68,2·27) |
| - Richest | Ref | Ref | Ref |
| Level of education |  |  |  |
| - No education | 2·95(1·37,6·34) | 2·53(0·82,7·79) | 1·25(0·27,5·80) |
| - Primary | 2·65(2·25,3·12) | 2·24(1·76,2·86) | 1·31(0·96,1·80) |
| - Secondary or higher | Ref | Ref | Ref |
| Area of residence |  |  |  |
| - Rural | 0·77(0·65,0·91) | 0·57(0·43,0·74) | 1·34(1·00,1·78) |
| - Urban | Ref | Ref | Ref |
| **Ethiopia** |  |  |  |
| Wealth quintile |  |  |  |
| - Poorest | 1·06(0·76,1·49) | 0·42(0·27,0·65) | 5·12(3·15,8·33) |
| - Poorer | 0·98(0·70,1·38) | 0·7(0·47,1·05) | 2·27(1·38,3·73) |
| - Middle | 1·35(0·97,1·87) | 1·2(0·83,1·73) | 1·31(0·82,2·10) |
| - Richer | 1·17(0·85,1·61) | 1·23(0·86,1·75) | 0·76(0·46,1·25) |
| - Richest | Ref | Ref | Ref |
| Level of education |  |  |  |
| - No education | 2·99(2·27,3·94) | 2·03(1·39,2·96) | 2·14(1·29,3·55) |
| - Primary | 1·41(1·09,1·83) | 1·06(0·71,1·58) | 1·64(0·99,2·73) |
| - Secondary or higher | Ref | Ref | Ref |
| Area of residence |  |  |  |
| - Rural | 1·84(1·33,2·54) | 1·65(1·21,2·23) | 1·67(1·12,2·49) |
| - Urban | Ref | Ref | Ref |
| **Ghana** |  |  |  |
| Wealth quintile |  |  |  |
| - Poorest | 3·3(1·79,6·09) | 4·59(1·94,10·9) | 0·56(0·19,1·65) |
| - Poorer | 4·11(2·25,7·51) | 3·7(1·51,9·06) | 1·07(0·35,3·24) |
| - Middle | 3·86(2·16,6·89) | 4·01(1·67,9·63) | 0·88(0·29,2·70) |
| - Richer | 2·94(1·65,5·21) | 4·45(1·91,10·4) | 0·46(0·14,1·44) |
| - Richest | Ref | Ref | Ref |
| Level of education |  |  |  |
| - No education | 2·42(1·64,3·57) | 2·41(1·45,4·03) | 0·94(0·44,2·00) |
| - Primary | 1·87(1·42,2·46) | 2·5(1·52,4·11) | 0·65(0·36,1·17) |
| - Secondary or higher | Ref | Ref | Ref |
| Area of residence |  |  |  |
| - Rural | 1·26(0·89,1·78) | 1·49(0·85,2·60) | 0·91(0·50,1·65) |
| - Urban | Ref | Ref | Ref |
| **Guatemala** |  |  |  |
| Wealth quintile |  |  |  |
| - Poorest | 1·52(1·17,1·98) | 1·76(1·01,3·09) | 0·86(0·47,1·55) |
| - Poorer | 1·48(1·15,1·90) | 1·66(0·94,2·94) | 0·9(0·49,1·65) |
| - Middle | 1·39(1·09,1·77) | 1·48(0·84,2·62) | 0·96(0·52,1·77) |
| - Richer | 1·25(0·98,1·59) | 1·45(0·80,2·62) | 0·85(0·44,1·61) |
| - Richest | Ref | Ref | Ref |
| Level of education |  |  |  |
| - No education | 4·5(3·50,5·80) | 5·34(3·40,8·38) | 0·87(0·51,1·50) |
| - Primary | 2·35(2·03,2·71) | 3·14(2·15,4·60) | 0·72(0·49,1·07) |
| - Secondary or higher | Ref | Ref | Ref |
| Area of residence |  |  |  |
| - Rural | 1·19(1·03,1·38) | 1·33(0·98,1·81) | 0·9(0·65,1·24) |
| - Urban | Ref | Ref | Ref |
| **Guinea** |  |  |  |
| Wealth quintile |  |  |  |
| - Poorest | 1·43(1·00,2·06) | 1·83(1·14,2·96) | 0·66(0·39,1·12) |
| - Poorer | 1·49(1·04,2·12) | 1·82(1·11,2·97) | 0·72(0·42,1·23) |
| - Middle | 1·28(0·91,1·79) | 1·42(0·89,2·26) | 0·83(0·49,1·40) |
| - Richer | 1·24(0·96,1·61) | 1·17(0·78,1·75) | 1·08(0·66,1·77) |
| - Richest | Ref | Ref | Ref |
| Level of education |  |  |  |
| - No education | 2·94(2·29,3·76) | 5·56(3·15,9·80) | 0·45(0·24,0·83) |
| - Primary | 1·87(1·43,2·44) | 2·43(1·32,4·48) | 0·8(0·41,1·58) |
| - Secondary or higher | Ref | Ref | Ref |
| Area of residence |  |  |  |
| - Rural | 1·45(1·10,1·92) | 1·65(1·16,2·34) | 0·87(0·59,1·27) |
| - Urban | Ref | Ref | Ref |
| **Haiti** |  |  |  |
| Wealth quintile |  |  |  |
| - Poorest | 2·52(1·74,3·65) | 2·08(1·30,3·31) | 1·36(0·74,2·50) |
| - Poorer | 2·06(1·43,2·97) | 1·78(1·11,2·86) | 1·23(0·66,2·30) |
| - Middle | 2·26(1·61,3·17) | 2·31(1·47,3·62) | 0·93(0·50,1·73) |
| - Richer | 1·92(1·41,2·61) | 2·31(1·56,3·43) | 0·61(0·33,1·15) |
| - Richest | Ref | Ref | Ref |
| Level of education |  |  |  |
| - No education | 6·13(4·40,8·52) | 5·02(3·32,7·57) | 1·22(0·60,2·48) |
| - Primary | 2·03(1·67,2·47) | 1·53(1·13,2·05) | 1·59(1·09,2·31) |
| - Secondary or higher | Ref | Ref | Ref |
| Area of residence |  |  |  |
| - Rural | 0·98(0·77,1·25) | 0·9(0·66,1·22) | 1·17(0·81,1·67) |
| - Urban | Ref | Ref | Ref |
| **Kenya** |  |  |  |
| Wealth quintile |  |  |  |
| - Poorest | 2·54(1·97,3·26) | 2·3(1·52,3·50) | 1·16(0·71,1·90) |
| - Poorer | 2·08(1·63,2·67) | 2·02(1·35,3·01) | 1·11(0·69,1·80) |
| - Middle | 2·3(1·80,2·94) | 1·93(1·29,2·88) | 1·34(0·82,2·17) |
| - Richer | 1·82(1·43,2·32) | 1·7(1·13,2·57) | 1·13(0·69,1·87) |
| - Richest | Ref | Ref | Ref |
| Level of education |  |  |  |
| - No education | 2·93(2·23,3·86) | 4·52(2·39,8·55) | 0·57(0·28,1·14) |
| - Primary | 2·12(1·84,2·45) | 2·37(1·68,3·36) | 0·87(0·60,1·27) |
| - Secondary or higher | Ref | Ref | Ref |
| Area of residence |  |  |  |
| - Rural | 0·68(0·58,0·80) | 0·79(0·56,1·13) | 0·94(0·65,1·38) |
| - Urban | Ref | Ref | Ref |
| **Malawi** |  |  |  |
| Wealth quintile |  |  |  |
| - Poorest | 2·99(2·47,3·63) | 1·78(1·36,2·35) | 2·31(1·66,3·22) |
| - Poorer | 2·27(1·88,2·74) | 1·37(1·04,1·80) | 2·23(1·60,3·10) |
| - Middle | 2·19(1·82,2·63) | 1·96(1·50,2·57) | 1·19(0·86,1·66) |
| - Richer | 1·81(1·52,2·16) | 1·58(1·22,2·05) | 1·25(0·90,1·74) |
| - Richest | Ref | Ref | Ref |
| Level of education |  |  |  |
| - No education | 4·03(3·07,5·29) | 3·92(2·66,5·78) | 1·12(0·65,1·95) |
| - Primary | 1·81(1·57,2·08) | 1·81(1·39,2·36) | 1·04(0·77,1·41) |
| - Secondary or higher | Ref | Ref | Ref |
| Area of residence |  |  |  |
| - Rural | 0·74(0·63,0·87) | 0·75(0·60,0·94) | 1·16(0·88,1·52) |
| - Urban | Ref | Ref | Ref |
| **Mali** |  |  |  |
| Wealth quintile |  |  |  |
| - Poorest | 0·95(0·72,1·26) | 1·28(0·90,1·81) | 0·49(0·32,0·77) |
| - Poorer | 1·36(1·02,1·80) | 1·71(1·21,2·40) | 0·59(0·38,0·90) |
| - Middle | 1·3(0·99,1·71) | 1·48(1·06,2·05) | 0·72(0·48,1·08) |
| - Richer | 1·25(0·99,1·58) | 1·25(0·92,1·69) | 0·98(0·66,1·46) |
| - Richest | Ref | Ref | Ref |
| Level of education |  |  |  |
| - No education | 2·77(2·27,3·38) | 3·7(2·64,5·19) | 0·66(0·44,0·99) |
| - Primary | 1·71(1·35,2·16) | 1·95(1·31,2·91) | 0·9(0·55,1·46) |
| - Secondary or higher | Ref | Ref | Ref |
| Area of residence |  |  |  |
| - Rural | 1·68(1·35,2·10) | 2·25(1·76,2·87) | 0·77(0·57,1·05) |
| - Urban | Ref | Ref | Ref |
| **Nigeria** |  |  |  |
| Wealth quintile |  |  |  |
| - Poorest | 1·58(1·18,2·12) | 0·75(0·45,1·24) | 2·81(1·58,5·01) |
| - Poorer | 2·37(1·80,3·13) | 1·05(0·64,1·72) | 3·01(1·71,5·30) |
| - Middle | 2·03(1·56,2·64) | 1·01(0·63,1·63) | 2·61(1·49,4·57) |
| - Richer | 1·92(1·48,2·49) | 1·25(0·79,1·99) | 1·85(1·06,3·25) |
| - Richest | Ref | Ref | Ref |
| Level of education |  |  |  |
| - No education | 7·08(6·04,8·30) | 9·74(6·89,13·8) | 0·66(0·45,0·96) |
| - Primary | 2·49(2·07,2·99) | 2·74(1·89,3·97) | 0·93(0·61,1·41) |
| - Secondary or higher | Ref | Ref | Ref |
| Area of residence |  |  |  |
| - Rural | 1·53(1·30,1·81) | 1·24(0·90,1·70) | 1·42(1·00,2·02) |
| - Urban | Ref | Ref | Ref |
| **Philippines** |  |  |  |
| Wealth quintile |  |  |  |
| - Poorest | 5·95(4·02,8·81) | 11·4(5·80,22·4) | 0·36(0·16,0·80) |
| - Poorer | 4·5(3·05,6·64) | 9·19(4·69,18·0) | 0·33(0·14,0·73) |
| - Middle | 3·43(2·31,5·10) | 4·59(2·26,9·30) | 0·6(0·26,1·39) |
| - Richer | 2·35(1·55,3·56) | 4·2(2·06,8·54) | 0·38(0·16,0·90) |
| - Richest | Ref | Ref | Ref |
| Level of education |  |  |  |
| - No education | 2·06(0·85,5·01) | 1·42(0·44,4·61) | 1·89(0·32,11·2) |
| - Primary | 3·43(2·76,4·25) | 2·45(1·80,3·33) | 1·73(1·15,2·61) |
| - Secondary or higher | Ref | Ref | Ref |
| Area of residence |  |  |  |
| - Rural | 0·99(0·81,1·23) | 1·17(0·84,1·62) | 0·76(0·51,1·14) |
| - Urban | Ref | Ref | Ref |
| **Rwanda** |  |  |  |
| Wealth quintile |  |  |  |
| - Poorest | 1·92(1·30,2·82) | 1·53(0·95,2·46) | 1·62(0·89,2·95) |
| - Poorer | 1·97(1·29,2·99) | 1·87(1·05,3·32) | 1·12(0·56,2·24) |
| - Middle | 1·64(1·07,2·50) | 1·27(0·70,2·30) | 1·55(0·76,3·18) |
| - Richer | 1·55(1·03,2·32) | 1·66(0·97,2·82) | 0·91(0·46,1·81) |
| - Richest | Ref | Ref | Ref |
| Level of education |  |  |  |
| - No education | 3·66(2·34,5·73) | 2·96(1·52,5·77) | 1·22(0·37,4·09) |
| - Primary | 2(1·48,2·70) | 1·6(0·88,2·91) | 1·34(0·68,2·65) |
| - Secondary or higher | Ref | Ref | Ref |
| Area of residence |  |  |  |
| - Rural | 0·56(0·40,0·78) | 0·59(0·40,0·88) | 1·14(0·71,1·84) |
| - Urban | Ref | Ref | Ref |
| **Senegal** |  |  |  |
| Wealth quintile |  |  |  |
| - Poorest | 4·8(3·43,6·71) | 4·91(3·09,7·78) | 0·98(0·53,1·80) |
| - Poorer | 3·45(2·48,4·79) | 3·2(2·03,5·04) | 1·14(0·62,2·09) |
| - Middle | 2·52(1·83,3·46) | 2·48(1·57,3·91) | 1·03(0·56,1·92) |
| - Richer | 1·54(1·10,2·14) | 1·47(0·92,2·35) | 1·09(0·57,2·10) |
| - Richest | Ref | Ref | Ref |
| Level of education |  |  |  |
| - No education | 2·9(2·37,3·53) | 3·64(2·04,6·49) | 0·8(0·44,1·47) |
| - Primary | 1·69(1·38,2·07) | 2·52(1·39,4·57) | 0·6(0·32,1·14) |
| - Secondary or higher | Ref | Ref | Ref |
| Area of residence |  |  |  |
| - Rural | 1·08(0·89,1·32) | 1·35(1·00,1·81) | 0·73(0·52,1·02) |
| - Urban | Ref | Ref | Ref |
| **South Africa** |  |  |  |
| Wealth quintile |  |  |  |
| - Poorest | 4·45(2·83,7·00) | 4·78(2·71,8·44) | 0·78(0·34,1·80) |
| - Poorer | 4·87(3·13,7·58) | 5·2(2·99,9·06) | 0·81(0·35,1·86) |
| - Middle | 3(1·95,4·61) | 3·13(1·81,5·42) | 0·86(0·37,2·04) |
| - Richer | 2·48(1·61,3·80) | 3·11(1·81,5·36) | 0·51(0·21,1·25) |
| - Richest | Ref | Ref | Ref |
| Level of education |  |  |  |
| - No education | 2·54(0·97,6·67) | 3·31(1·18,9·30) | 1(1,1) |
| - Primary | 1·04(0·83,1·30) | 1·01(0·78,1·31) | 1·13(0·65,1·95) |
| - Secondary or higher | Ref | Ref | Ref |
| Area of residence |  |  |  |
| - Rural | 1·02(0·81,1·27) | 1·14(0·86,1·50) | 0·81(0·56,1·18) |
| - Urban | Ref | Ref | Ref |
| **Tanzania** |  |  |  |
| Wealth quintile |  |  |  |
| - Poorest | 3·4(2·44,4·75) | 2·39(1·38,4·15) | 1·73(0·95,3·16) |
| - Poorer | 3·2(2·28,4·49) | 1·88(1·03,3·43) | 2·14(1·12,4·12) |
| - Middle | 2·72(1·96,3·76) | 2·53(1·49,4·29) | 1·17(0·65,2·11) |
| - Richer | 2·23(1·68,2·97) | 2·59(1·55,4·33) | 0·85(0·47,1·52) |
| - Richest | Ref | Ref | Ref |
| Level of education |  |  |  |
| - No education | 8·2(5·87,11·4) | 4·77(2·22,10·2) | 1·68(0·72,3·92) |
| - Primary | 3·94(3·11,4·99) | 2·05(1·01,4·13) | 2·05(0·98,4·29) |
| - Secondary or higher | Ref | Ref | Ref |
| Area of residence |  |  |  |
| - Rural | 0·7(0·54,0·91) | 0·6(0·40,0·91) | 1·56(1·00,2·44) |
| - Urban | Ref | Ref | Ref |
| **Uganda** |  |  |  |
| Wealth quintile |  |  |  |
| - Poorest | 2·48(1·92,3·20) | 2·72(1·84,4·03) | 0·93(0·60,1·44) |
| - Poorer | 2·28(1·78,2·92) | 2(1·36,2·95) | 1·25(0·80,1·94) |
| - Middle | 1·73(1·35,2·22) | 1·46(0·99,2·15) | 1·33(0·84,2·09) |
| - Richer | 1·39(1·10,1·75) | 1·13(0·79,1·61) | 1·4(0·90,2·17) |
| - Richest | Ref | Ref | Ref |
| Level of education |  |  |  |
| - No education | 2·91(2·11,4·00) | 4·41(2·82,6·90) | 0·39(0·21,0·73) |
| - Primary | 1·7(1·45,2·00) | 1·78(1·34,2·37) | 0·94(0·68,1·31) |
| - Secondary or higher | Ref | Ref | Ref |
| Area of residence |  |  |  |
| - Rural | 0·93(0·76,1·15) | 0·81(0·61,1·09) | 1·35(0·96,1·91) |
| - Urban | Ref | Ref | Ref |
| **Zimbabwe** |  |  |  |
| Wealth quintile |  |  |  |
| - Poorest | 3·03(1·96,4·70) | 1·72(1·00,2·97) | 2·62(1·44,4·75) |
| - Poorer | 2·49(1·61,3·86) | 1·38(0·80,2·40) | 2·71(1·48,4·96) |
| - Middle | 2·62(1·70,4·03) | 1·49(0·86,2·57) | 2·52(1·39,4·58) |
| - Richer | 2·41(1·75,3·31) | 1·55(0·96,2·50) | 2·03(1·12,3·70) |
| - Richest | Ref | Ref | Ref |
| Level of education |  |  |  |
| - No education | 2·05(0·84,5·02) | 3·12(1·19,8·14) | 1(1,1) |
| - Primary | 2·05(1·70,2·48) | 1·95(1·49,2·56) | 1·09(0·76,1·56) |
| - Secondary or higher | Ref | Ref | Ref |
| Area of residence |  |  |  |
| - Rural | 1·09(0·77,1·53) | 0·92(0·63,1·35) | 1·84(1·23,2·76) |
| - Urban | Ref | Ref | Ref |

AOR: Adjusted odds ratio; CI: Confidence interval

AOR>1 (<1) represents increased (decreased) in the association between adolescent mother and social risk factors at endline compared to baseline
