# Supplementary material for: Transition in social risk factors and adolescent motherhood in low- income and middle- income countries: Evidence from Demographic and Health Survey data, 1996–2018
Source: PLOS Glob Public Health. 2022 May 11;2(5):e0000170. doi: 10.1371/journal.pgph.0000170 (PMC10021223; doi:10.1371/journal.pgph.0000170)
Supplement: Attachment — Submitted filename: Response to Reviewers.docx [file pgph.0000170.s006.docx]

**Response Letter**

16 December 2021

To:

The Editor

PLOS Global Public Health

Subject: Re-submitting the manuscript with reference number: PGPH-D-21-00062

Dear Editor,

We would like to thank you for giving us the opportunity to address editorial comments and re-submit above mentioned manuscript to your journal. Following the editor’s and reviewers’ advice, we have revised our manuscript.

We hope that the revised version has improved substantially comply the manuscript format. We have attached a point-by-point response to the review comments. In this submission, we have uploaded both the response to the editor and the revised manuscript (clean and track-changes version) for re-review.

We thank you again for the opportunity to revise our work again and look forward to receiving your reply.

Sincerely,

M. Mamun Huda

**Editor(s)' Comments to Author:**

Academic Editor
PLOS Global Public Health

Journal Requirements:

1. Please list the name and version of any software package used for statistical analysis, alongside any relevant references. For more information on PLOS ONE's expectations for statistical reporting, please see <https://journals.plos.org/plosone/s/submission-guidelines.#loc-statistical-reporting>.

**Response:** Name of version of the software is mentioned in the revised manuscript. Please see “statistical analysis section” on Page 9

2. Please update the completed 'Competing Interests' statement, including any COIs declared by your co-authors. If you have no competing interests to declare, please state "The authors have declared that no competing interests exist". Otherwise please declare all competing interests beginning with the statement "I have read the journal's policy and the authors of this manuscript have the following competing interests:"

**Response:** We do not have any competing of interest, which has been mention on page 20 in the revised manuscript as:

“**Competing interests**: The authors have no competing interests to declare.”

**Comments to the Author**

1. Does this manuscript meet PLOS Global Public Health’s [publication criteria](https://journals.plos.org/globalpublichealth/s/criteria-for-publication)? Is the manuscript technically sound, and do the data support the conclusions? The manuscript must describe methodologically and ethically rigorous research with conclusions that are appropriately drawn based on the data presented.

Reviewer #1: Yes

Reviewer #2: Partly

 2. Has the statistical analysis been performed appropriately and rigorously?

 Reviewer #1: Yes

Reviewer #2: Yes

 3. Have the authors made all data underlying the findings in their manuscript fully available (please refer to the Data Availability Statement at the start of the manuscript PDF file)?

The [PLOS Data policy](https://journals.plos.org/globalpublichealth/s/data-availability) requires authors to make all data underlying the findings described in their manuscript fully available without restriction, with rare exception. The data should be provided as part of the manuscript or its supporting information, or deposited to a public repository. For example, in addition to summary statistics, the data points behind means, medians and variance measures should be available. If there are restrictions on publicly sharing data—e.g. participant privacy or use of data from a third party—those must be specified.

 Reviewer #1: Yes

Reviewer #2: Yes

 4. Is the manuscript presented in an intelligible fashion and written in standard English?

PLOS Global Public Health does not copyedit accepted manuscripts, so the language in submitted articles must be clear, correct, and unambiguous. Any typographical or grammatical errors should be corrected at revision, so please note any specific errors here.

 Reviewer #1: Yes

Reviewer #2: No

 5. Review Comments to the Author

Please use the space provided to explain your answers to the questions above. You may also include additional comments for the author, including concerns about dual publication, research ethics, or publication ethics. (Please upload your review as an attachment if it exceeds 20,000 characters)

**Reviewer #1: Ganchimeg Togoobaatar**

**Comments to the Author:**

Reviewer #1: The manuscript titled "Transition in social risk factors and adolescent motherhood in low-and middle-income countries: Evidence from Demographic and Health Survey data , 1996-2018" is excellent work to describe changes of socioeconomic factors of the adolescent motherhood in developing countries. Hope findings of the study will have significant impact on prioritising women and adolescent health and taking action on reducing inequities in SDG eras.
I have no comments.
Please provide explains full meaning of abbreviation in line 86., SRHR and line 133- ICF

**Response:** Thank you so much for inspiring comments about our work. SRHS has been spelled out as sexual and reproduction health right in the revised version (page 4). ICF International, Inc. is a global consulting and technology service provider headquartered in in Virginia, USA. ICF was founded in 1969 as the Inner City Fund. The company reorganized as a consulting firm and renamed itself ICF Incorporated. ICF International rebranded as ICF in 2016. We have revised the sentence by mentioning what ICF is (Please see page 7).

**Reviewer #2: Mengjia Liang**

**Comments to the Author:**

Reviewer #2: The study explores association between adolescent motherhood and the social risk factors, as well as changes in the strength of the association over time in 20 LMICs using DHS data. The results constitute a significant addition to the field. However, a few methodological issues must be addressed before the manuscript is suitable for publication. Also, the authors could potentially benefit from editorial services. My comments on specific lines of the paper are as follows.

Lines 85-88: lack of sufficient interventions from the governments may be one of the reasons why high levels of adolescent motherhood have been observed in many LMICs but should not be the only reason. The authors should consider doing a more comprehensive review or refining the wording.

**Response:** Thank you for your suggestion. Following your suggestion, the section has been updated as:

*“………………………………* *Governments in many LMICs remain reluctant to move beyond the* *sexual and reproduction health right program of abstinence messaging for unmarried adolescents, and child marriage remains acceptable (or even encouraged) in many countries. Legal restrictions on emergency contraception and abortion in many LMICs are barriers to avoiding unwanted pregnancies (Center for Reproductive Rights 2015). Furthermore, adolescent friendly health systems for sexual health, family planning, and maternal health are lacking in many LMICs (Morris JL et al 2015). As such, the prevalence of adolescent motherhood in LMICs remains high.”*

Lines 89-106 discuss the risk factors of adolescent pregnancy at the individual level. The paper could be strengthened by including an overview of the risk factors at other levels, such as community and society levels.

**Response:** We appreciate the reviewer suggestion to discuss the community-level effect on the occurrence of adolescent motherhood. However, discussing community-level risk factors is beyond the scope as we are not assessing community effect in our study. However, several studies evaluated community effect on the prevalence of adolescent pregnancy and motherhood in the literature. The objective of our study is to see the transition of individual-level risk factors and their association with the prevalence of adolescent motherhood in LMICs. Therefore, our discussion primarily focused on the induvial level risk factor. However, following the reviewer suggestion, we acknowledged different levels of risk and protective factors of adolescent motherhood in the introduction of our revised version of the manuscript as:

*“…………. The social factors of adolescent pregnancy and motherhood may also operate at different levels such as individual, family or school, community, and national level (Cherry et al 2009, Magadi et al 2017). Factors that examined adolescent pregnancy and motherhood were mostly focused on the individual level, limitation on the family and community-level factors, and few studies on national or country contexts (Mmari et al. 2013, Review articles) in the literature.*”

Line 89: the authors introduce the notion “social risk factors” here. Would be good if the concept can be defined and a brief discussion on social risk factors vs. non-social risk factors can be provided.

**Response:** In literature, there are different types of risk factors for adolescent motherhood. However, discussing social vs non-social risk factors is not in the interest of this study. We assessed three individual-level factors in this study that fit under the umbrella of social factors among different types of risk factors in the literature. Following the reviewer’s suggestion, we have further revised this section to clarify social vs non-social risk factor issues in the introduction of our revised version of the manuscript as:

*“………….* *Understanding factors associated with the prevalence of adolescent motherhood in LMICs enables the design of more targeted prevention strategies. Factors related to the adolescent pregnancy and motherhood are well studied in many countries in LMIC. Those studies reported various types of risk and protective factors, including social (i.e., sociocultural, environmental, and economic factors), behavioural (sexual risk behaviour, excessive use of alcohol, substance abuse), and health service-related (i.e., inadequate, and unskilled health workers, long waiting time and lack of privacy at clinics, cost and misconceptions about contraceptives, and non-friendly adolescent reproductive services) factors, etc. The social factors of adolescent pregnancy and motherhood may also operate at different levels such as individual, family or school, community, and national level (Cherry et al 2009, Magadi et al 2017). Factors that examined adolescent pregnancy and motherhood were mostly focused on the individual level, limited focused on the family and community-level factors, and few studies focused on national or country contexts (Mmari et al. 2013, Review articles) in the literature.*”

Lines 130-131: The authors indicate that data from 20 LMICs are used in the analysis. However, it is unclear what the selection criteria are.

**Response:** The selection criterion for selecting the 20 LIMCS were mentioned end of the 2^nd^ paragraph in “Data source and participants”. We also referred the supplementary figure (S1 Fig) so that where it was shown how the analytic sample were selected. However, based on the reviewer concern we revised both the text and supplementary figure (S1 fig). Please see the text and supplementary figure 1 in the revised manuscript.

*“…. The sample selection criterion was, country with at least two all-women surveys: a survey in 1996-2003, near to MDG started (as baseline) and another survey in 2014-2018, recent time (as endline) so that the duration for assessing the transition of social factors can be nearly 15 years for all the studied countries.”*

Lines 136-137: Would be good if the authors can clarify what regions are they - are these SDG regions?

**Response:** These are geographic regions across different continents. DHS classifies survey countries into six different geographic regions: 1) Sub-Saharan Africa, 2) North Africa/West Asia/ Europe, 3) Central Asia, 4) South & Southeast Asia, 5) Oceania, and 6) Latin America & Caribbean. For clarity, we have replaced "regions" with “geographic regions” in the revised manuscript. Please see page 7 in the revised manuscript.

Lines 152-158; 172-173: The authors assesse changes in household socioeconomic status using the household wealth index. However, the DHS wealth index is intended to compare households within a single survey. Scores are relative, not absolute; in any particular survey, the mean wealth score is 0. As a country's population's economic status improves and household services (such as piped drinking water) and household assets (such as televisions) become more affordable, the combination of assets and services that ranks a household in the top quintile in an earlier survey may rank a household in the bottom quintile in a later survey. See further details in references below. The implications and limitations of using the wealth index must be discussed in addition to lines 361-366.

References:
Staveteig, Sarah and Lindsay Mallick. 2014. Intertemporal Comparisons of Wealth with DHS Data: A Harmonized Asset Index Approach. DHS Methodological Reports No. 15. Rockville, Maryland, USA: ICF International.
Rutstein, Shea O. and Kiersten Johnson. 2004. The DHS Wealth Index. DHS Comparative Reports No. 6. Calverton, Maryland: ORC Macro.

**Response:** We agree with the reviewer that the wealth index is the relative, not an absolute measure. Thus, assessing trends in a specific socio-economic status (e.g., poorest) defined by the DHS wealth index) in a population should be problematic. In this study, we were not interested to see the trends in the proportion of a specific socio-economic status in the population of the studied countries, rather assessed how odds (an indirect measure of risk) of adolescent motherhood being a poorest (lowest quintile) adolescent has changed during the study period compared to their counterpart, richest (highest quintile) adolescent girls. That is, we estimated the difference in odds ratio (poorest vs richest) between baseline and endline surveys. Therefore, the comparator (richest) in the risk estimation for the poorest was always at the same time point, not the other time point. To estimate this type of change is the odds ratio, we used an interaction regression model with an interaction term of time (baseline vs endline) and wealth quintile (e. g., poorest vs richest), mutually adjusted by other social factors.

In addition to model estimates of the changes in risk factor, we also reported the crude proportion of different wealth quintiles among adolescent mothers and non-mothers at both baseline and endline, as well as the difference in the proportion between baseline and endline separately for mother and non-months to understand the changes (trends) of those wealth quintiles in the studied countries. This trend might be misleading because of the relative measure of the wealth index, as the reviewer pointed out. We also acknowledged this limitation in discussion as (Please see page 18):

*“……Thirdly, unlike the measurement of the level of education and areas of residence, the wealth quintile is a household measure of wealth relative to the wealth of other households within the survey and is only a proxy measure of adolescent girls’ socioeconomic status. Thus, the wealth quintile measured at baseline and endline survey may not give us the appropriate trend of adolescent girls with a particular level of wealth quintile. To overcome this limitation, in addition to examine the changes in a specific quintile (poorest), we also examined the changes in the difference between first (poorest) and last (richest) quantile during the study period in this study”*

As already mentioned in the limitation, to overcome this problem, we also observed changes with respect to the comparative group (richest) at the same time points which was the difference in the proportion of poorest and richest (which was reported as ‘gap’ but revised as ‘difference’ in the revised manuscript) and how this difference/gap has changed over time. This was described in the results as (Please see page 10):

*“……. Over time, the concentration in the richest wealth quintile of adolescent non-mothers decreased (by -6·26%, 95%CI -6·85 to -5·67) and the concentration in the poorest of adolescent mothers increased (by 5·87%, 95% CI 4·74 to 7·00). The difference between the richest and poorest narrowed over time for the non-mothers (by 7.24%). For the mothers, the gap between richest and poorest widened by 12·22%, with an accelerated concentration of the mothers shifting down the wealth quintiles (22·66% to 28·53% of mothers in poorest households) and fewer mothers remaining in the richest quintiles (16·66% to 10·31% of mothers in the richest households over time) (Table 1 and S2 Fig.). “*

Lines 185: Suggest to the authors to further elaborate “cluster/cluster levels”.

**Response:** Following receiver’s suggestion we have elaborated “cluster/cluster levels” in method section.

We added the following text in the “Data source, study design and participants” section on page 6 to clarify a cluster.

*“……….* *In DHS, usually, countries were divided into sub-national regions mostly based on local administrative boundaries. In each region, the population were further stratified according to urban and rural area residence (known as strata). Within these strata, enumeration areas mostly known as clusters were identified based on the most recent population census. At the first stage, these primary sampling units were selected based on probability proportional to the population size from each stratum. Complete household listing was made for each of the selected clusters. At the second stage, approximately 30-40 and 20-25 households were selected by equal probability systematic sampling in the selected clusters from rural and urban areas respectively.”*

We also added the following text in the “Statistical analysis” section on page 9 to clarify the cluster level.

*“…………. In the DHS survey design (multistage cluster sampling), interview participants are usually nested within each country's survey cluster (at a higher level). When we pooled the study countries for our pooled analysis, clusters were further nested within countries. Finally, in the pooled analysis, study participants were nested at three hierarchical levels: country, cluster, and individual. Due to this hierarchical structure of the data, study participants who nested at different levels correlate with one another. Failing to address the correlation may underestimates the standard error, leading to an overstatement of statistical significance. The multilevel regression model can handle this type of correlated data and produce unbiased estimates”*

Lines 201-202: The statement does not seem to be accurate considering improved education and urbanization.

**Response:** We found that the proportion of adolescent mothers with the lowest wealth quintile (poorest) has increased at 5.87% (see line 243 and the last column in Table 1), whereas the proportion of adolescent mothers without formal education has decreased (the last column in Table 1) at endline compared to baseline. Therefore, the change direction in the proportion of the three social factors was not the same in this study.

Lines 205-212: Further elaborations on “gaps between the richest and the poorest” can be helpful. The authors may wish to explore the measures “% mothers/non-mothers among poorest/richest”.

**Response:** In the revised version, we replaced the word ‘gap’ with ‘difference’ (see revised text in page 10 and table 1). We meant gap as difference, that is, the difference between the richest and the poorest. Larger the differences in the proportion between the richest and the poorest represent the higher disparities between these two groups and vice versa.

Our intention in this descriptive analysis was to see how the proportion of all studied social factors has changed for mothers and non-mothers over time (Table 1). However, our regression model has already addressed the author's advice (measuring % mothers/non-mothers among social factors”). We estimated the odds of being a mother among adolescent girls having different social backgrounds (e.g., poorest vs richest) and how the odds ratio has changed over time.

Lines 224-229: When discussing changes between the two surveys, it would be helpful to know the intervals between the surveys. If the intervals have a large range, the authors should discuss the implications and consider taking this factor in the analysis. In addition, the authors should consider if additional surveys were done between the baseline and endline surveys, and offer an explanation for why these surveys were not included in the study.

**Response:** Baseline and endline survey year has been mentioned in the text while discussing the country-specific results. Average during has been mentioned in method (line 170, page 7) and supplementary figure 1.

It would be better if we could consider all the surveys between baseline and endline surveys. However, in-between surveys highly varied across different studied countries. For example, South Africa had no in-between survey, Chan and Colombia had only one in-between survey. In contrast, some other countries had many in-between surveys (e.g. Senegal had six surveys). Thus for the consistency in the number of surveys, only two surveys (baseline and endline) were included in the study. We have mentioned this in our revised manuscript as (line 171, page 7):

*“…. For the 20 selected countries, the number of surveys highly varied between baseline and endline surveys. Thus, for the consistency in the number of surveys, only two surveys (baseline and endline) were included in the study.”*

Lines 238-240: The rationale for a threshold of 22% is unclear. Would be great if the authors could clarify.

**Response:** 22% is the global/overall mean prevalence of adolescent motherhood in the study countries, at baseline. We revised the figure 1 indicating global mean prevalence of adolescent motherhood at baseline and endline. Text in the result section also revised as:

*“………..That is, in this region, baseline prevalence was greater than global mean prevalence of the studied countries, 22% for 6/8 countries that experienced a decrease in adolescent motherhood and was greater than 22% for 2/8 countries that experienced no change in adolescent motherhood (Fig 1 and S1 Table). “*

Line 335: Typo – “studies” should be “countries”

**Response:** Corrected. Thank you.

Line 347 “Our findings on the increased trend of disadvantaged (poorest and living in a rural area) adolescent motherhood…” seems to be inconsistent with lines 217-219 “There is a higher concentration of adolescent mothers in rural areas (72·24% at endline) than non-mothers (59·11% at endline), and this changed little over time (the change is not significant)”. Would be good if the authors can clarify.

**Response:** Corrected by removing rural areas (please see line 386, page 18)

*“Our findings on the increased trend of disadvantaged (poorest) adolescent motherhood…”*

Lines 352-356: The paper could benefit from a more elaborated discussion on these points instead of a general statement.

Response: Unfortunately, we could not follow which discussion the reviewer is asking to elaborate on. In the submitted manuscript, these lines belong to text related to limitations and part of the conclusions in the discussion. If the reviewer indicates the limitation of the relative measure of wealth index, we discussed this issue in lines 345 to 352 (lines 404-410 in revised manuscript). We also made a detailed response on this issue against the reviewer’s another comment above.

Lines 368-370: Common quality issues on pregnancy/birth data in DHS should also be discussed.

**Response:** We have updated limitation section considering reviewer’s suggestion as:

*“…………**Fourth, accuracy of the age of the participants at the time of interview and the year of their first birth can be affected by recall bias, which might have introduced bias into the prevalence estimate Fifth, our estimates included livebirths and current pregnancies, but did not include stillbirths, miscarriages, or abortions.* *Data on stillbirths, miscarriages, or abortions are not consistently reported in all the countries. Thus, in this study, the focus is on motherhood rather than pregnancy.”*

Lines 376-377: The statement “These results indicate that little has been done to improve the prevalence of adolescent motherhood in LMICs…” does not seem to be accurate. The results show that half of the countries in the study have experienced a decrease in the prevalence of adolescent motherhood. Also, much work has been done in the field, the lack of progress in some countries might be attributed to a variety of factors. Absolute assertions like these should be avoided by the authors.

**Response:** We have revised the conclusion (See line 413-425, page 19):

*“……These results indicate that more effort is needed to improve the prevalence of adolescent motherhood in LMICs. “*

6. PLOS authors have the option to publish the peer review history of their article ([what does this mean?](https://journals.plos.org/globalpublichealth/s/editorial-and-peer-review-process#loc-peer-review-history)). If published, this will include your full peer review and any attached files.


**Do you want your identity to be public for this peer review?** If you choose “no”, your identity will remain anonymous but your review may still be made public.


For information about this choice, including consent withdrawal, please see our [Privacy Policy](https://www.plos.org/privacy-policy).

Reviewer #1: **Yes:** Ganchimeg Togoobaatar

Reviewer #2: **Yes:** Mengjia Liang

[NOTE: If reviewer comments were submitted as an attachment file, they will be attached to this email and accessible via the submission site. Please log into your account, locate the manuscript record, and check for the action link "View Attachments". If this link does not appear, there are no attachment files.]

While revising your submission, please upload your figure files to the Preflight Analysis and Conversion Engine (PACE) digital diagnostic tool, <https://pacev2.apexcovantage.com/>. PACE helps ensure that figures meet PLOS requirements. To use PACE, you must first register as a user. Registration is free. Then, login and navigate to the UPLOAD tab, where you will find detailed instructions on how to use the tool. If you encounter any issues or have any questions when using PACE, please email PLOS at [figures@plos.org](mailto:figures@plos.org). Please note that Supporting Information files do not need this step.

*In compliance with data protection regulations, you may request that we remove your personal registration details at any time.* [*(Remove my information/details)*](https://www.editorialmanager.com/pgph/login.asp?a=r)*. Please contact the publication office if you have any questions.*
